# Metformin-Induced Hemolysis in a Patient With Glucose-6-Phosphate Dehydrogenase Deficiency Presenting With Concurrent Idiopathic Steven-Johnson Syndrome/Toxic Epidermal Necrolysis

**DOI:** 10.7759/cureus.18506

**Published:** 2021-10-05

**Authors:** Avijoy Roy Choudhury, Cecilia Gadaga, Layuren Moodley, Aru Moodley

**Affiliations:** 1 Internal Medicine, The University of Western Australia (UWA) Medical School, Perth, AUS; 2 Internal Medicine, Texila American University, Texas, USA; 3 Internal Medicine, Xavier University School of Medicine, Oranjestad, ABW; 4 Family Medicine, Western Australian General Practice Education and Training (WAGPET), Bentley, AUS; 5 Medicine, The University of Notre Dame Australia, Fremantle, AUS

**Keywords:** glucose-6-phosphate-dehydrogenase deficiency (g6pd), : metformin, hemolytic anemia, toxic epidermal necrolysis (ten), steven johnson syndrome

## Abstract

Metformin is one of the most widely prescribed medications for type 2 diabetes. While extremely rare, metformin has been reported to cause hemolysis in patients with glucose-6-phosphate dehydrogenase deficiency. In this paper, we present a case of a patient with previously undiagnosed glucose-6-phosphate dehydrogenase deficiency who presented with hemolysis likely induced by metformin. The patient concurrently presented with idiopathic Steven-Johnson syndrome/toxic epidermal necrolysis (SJS/TEN). Metformin causing hemolysis is extremely rare but considering the severe outcomes, it is something that medical practitioners need to be aware of.

## Introduction

Metformin is an oral medication that is commonly used in the treatment of type 2 diabetes mellitus. The common adverse effects of metformin include gastrointestinal symptoms, vital B12 deficiency, and lactic acidosis [1]. That being said, in patients with glucose-6-phosphate dehydrogenase (G6PD) deficiency, hemolytic anemia is a rare complication that may arise [2,3]. In this case report, we outline a patient with a previously undiagnosed G6PD deficiency that had an episode of hemolytic anemia following the administration of metformin. Furthermore, the patient had a concurrent episode of idiopathic Steven-Johnson syndrome/toxic epidermal necrolysis (SJS/TEN). This report aims to alert that metformin can induce hemolysis in patients with G6PD deficiency. Furthermore, this report also aims to emphasize the importance of investigating G6PD deficiency in patients presenting with hemolytic anemia.

## Case presentation

A 35-year-old African American female with a past medical history of type 2 diabetes mellitus, hypertension, and iron-deficient anemia presented to the hospital with fever, chills, and a diffuse erythematous rash which got worse after taking her current usual medications which are metformin, losartan, and simvastatin. The patient had been taking metformin, losartan, and simvastatin for greater than 12 months. Approximately 12 hours following the initial presentation, the erythematous rash became more extensive and skin peeling was present. Nikolsky sign was positive. Mucosal membrane involvement was present with severe conjunctivitis characterized by conjunctival injections and photophobia.

Comprehensive investigations were conducted for this patient which included a complete blood count. The patient had lab results conducted eight weeks prior by her primary care physician. The lab results from eight weeks prior showed the patient’s hemoglobin as 12.9 g/dL and her platelet count as 125,000 mm^3^. In comparison to those results, the patient had drastic falls in her hemoglobin and platelet levels on initial presentation as her hemoglobin was 10.0 g/dL and her platelet count was 90,000 mm^3^. Furthermore, following the administration of the patient’s regular medications, there was an even further fall in the patient’s hemoglobin and platelet levels. These results showed a hemoglobin level of 8.2 g/dL and a platelet level of 60,000 mm^3^ (Table 1). A direct Coombs test was conducted, which was negative ruling out Evans Syndrome. Furthermore, an indirect Coombs test was conducted which positive thus suggested that hemolysis was present. Furthermore, bilirubin levels were increased from eight weeks prior to initial presentation and further increased during lab results completed following administration of the patient's regular medications (Table 1). The results from other blood labs including lipid screen, glucose, HbA1C, urea, electrolytes, and creatinine were stable. 

**Table 1:** Blood laboratory results of the patient

	8 Weeks Prior to Admission	At Admission	Following Administration of Regular Medication
Na^+^	141 mEq/L	136 mEq/L	140 mEq/L
K^+^	4.5 mEq/L	4.3 mEq/L	4.3 mEq/L
Cl^-^	101 mEq/L	100 mEq/L	107 mEq/L
Glucose (serum)	81 mg/dL	93 mg/dL	89 mg/dL
Bilirubin (total)	2.1 mg/dL	3.8 mg/dL	7.0 mg/dL
Creatinine (serum)	1.0 mg/dL	1.1 mg/dL	0/87 mg/dL
Blood Urea Nitrogen	12.2 mg/dL	13.0 mg/dL	12.7 mg/dL
Cholesterol	150 mg/dL	155 mg/dL	146 mg/dL
Triglycerides	92 mg/dL	89 mg/dL	95 mg/dL
Hemoglobin	12.9 g/dL	10.0 g/dL	8.2 g/dL
Mean Corpuscular Volume	69.2 μm^3^	68.8 μm^3^	68.8 μm^3^
White Blood Cell Count	8,900/mm^3^	16,000/mm^3^	16.000/mm^3^
Platelet Count	125,000/mm^3^	90,000/mm^3^	60,000/mm^3^

Further investigating the patient’s hemolysis, testing was conducted which showed that the patient had a G6PD deficiency with a level of less than 8.8 units per gram of hemoglobin. Following this finding, the hemolysis was diagnosed as metformin-induced hemolytic anemia in a patient with G6PD deficiency. The metformin was ceased and the patient's hemolytic anemia improved. 

Looking at the erythematous rash, SJS with an overlap of TEN was suspected resulting in detailed differential analysis. The patient denied taking any sulfur-containing drugs, sulfate antibiotics, or hydralazine. The patient did not have any porphyria as per the uroporphyrinogen test and the paraneoplastic of the young was statistically unlikely. Herpes simplex virus screen, hepatitis C virus screen, and mycoplasma screen were all negative. Streptococcal scalded skin syndrome was ruled out as presentation had mucosal involvement with conjunctivitis. The patient had no history of malignancy and there was no family history of SJS or TEN. Furthermore, the patient had no immunosuppressive conditions and was taking no immunosuppressive medication. Following a thorough investigation, idiopathic SJS/TEN was diagnosed.

In treating the SJS/TEN overlap, the patient was stabilized and the patient underwent skin grafts to treat the condition.

## Discussion

Metformin is a biguanide and the first-line pharmacological treatment for type 2 diabetes mellitus. The drug acts on the liver where it inhibits gluconeogenesis while also having insulin sensitivity enhancement effects [1]. Common adverse effects of metformin include gastrointestinal symptoms such as mild anorexia, nausea, abdominal discomfort, and diarrhea. Vitamin B12 deficiency is also an adverse effect of metformin while lactic acidosis is a rare, but potentially fatal, complication [4].

In patients with G6PD deficiency, there have been reports of metformin inducing hemolytic anemia. G6PD deficiency is an X-linked recessive disorder resulting in an inborn error of metabolism that predisposes to the breakdown of red blood cells [5]. Hemolysis in patients with G6PD deficiency is commonly caused by bacterial or viral infections. Many drugs also induce hemolysis in patients with G6PD deficiency with the common drugs being dapsone, flutamide, mafenide cream, methylene blue, nalidixic acid, nitrofurantoin, phenazopyridine, primaquine, rasburicase, sulfacetamide, sulfamethoxazole, and sulfanilamide. Fava beans are also a common trigger for hemolysis in G6PD deficient patients. Triggers such as certain drugs, fava beans, or infections result in the build-up of reactive oxygen species inside the cell and with a deficiency of G6PD, these reactive oxygen species cannot be broken down. This build-up of reactive oxygen species results in hemolysis [6].

The incidence of metformin inducing hemolytic anemia in G6PD deficient patients is very low, but it has been reported in case reports in the past. A common feature in the majority of the reports is that the hemolysis took place within the first 12 days of starting metformin [2,3]. Developing an undeniable diagnosis of metformin causing hemolysis is impossible but, in this case, considering the lack of commonly known triggers, it is a likely cause of the hemolytic anemia. Although very rare, medical practitioners should be aware of metformin inducing hemolytic anemia as it can lead to serious and potentially fatal outcomes.

There have been no reports relating metformin administration causing SJS or TEN. A hypothesis was presented in 2007 which suggested G6PD deficiency could be a risk factor for the development of TEN but this was not followed further, and no further evidence has been presented on this concept [7]. SJS/TEN is commonly triggered by certain medications or infections. The common medications include allopurinol, nevirapine, aromatic antiseizure medication, and antibacterial sulfonamides while Mycoplasma pneumoniae is a common infectious cause [8,9]. That being said, over 33% of presentations of SJS or TEN have been idiopathic [10]. Considering this patient had no significant risk factors for SJS or TEN, such as HIV infection, systemic lupus erythematosus, malignancy, or family history of SJS/TENS, a definite cause could not be found. As a result, the diagnosis of idiopathic SJS/TEN was made.

## Conclusions

We have reported the case of a 35-year-old female with previously undiagnosed G6PD deficiency who presented with hemolysis likely related to the administration of metformin. Furthermore, this patient concurrently presented with idiopathic SJS/TEN. Hemolysis induced by metformin is extremely rare but it has been reported in the literature. Despite the rare nature of the occurrence, medical practitioners should be aware of this adverse effect of metformin as it has severe outcomes.
